# Impact of Perioperative Oral Care on the Length of Hospital Stay in Cardiovascular Surgery Patients

**DOI:** 10.3290/j.ohpd.c_2585

**Published:** 2026-04-13

**Authors:** Hiroshi Ishibashi, Yoshiharu Enomoto, Satoshi Fukuzawa, Keiji Tabuchi, Yasuyuki Suzuki, Toru Yanagawa

**Affiliations:** a Hiroshi Ishibashi PhD Student, Doctoral Program in Clinical Sciences, Graduate School of Comprehensive Human Sciences, University of Tsukuba, and Department of Cardiology, National Hospital Organization, Tokyo National Hospital. Analyzed the data and results, wrote the manuscript.; b Yoshiharu Enomoto Center Director, Department of Cardiovascular Surgery, Mito Saiseikai General Hospital, Mito, Japan. Wrote the manuscript.; c Satoshi Fukuzawa Lecturer, Department of Oral and Maxillofacial Surgery, Institute of Clinical Medicine, Faculty of Medicine, University of Tsukuba, Tsukuba, Japan. Wrote and reviewed the manuscript.; d Keiji Tabuchi Professor, Department of Otorhinolaryngology, Head and Neck Surgery, Institute of Clinical Medicine, Faculty of Medicine, University of Tsukuba, Tsukuba, Japan. Wrote and reviewed the manuscript.; e Yasuyuki Suzuki Professor, Department of Cardiovascular Surgery, Institute of Clinical Medicine, Faculty of Medicine, University of Tsukuba, and Department of Cardiovascular Surgery, Institute of Clinical Medicine, Faculty of Medicine, University of Tsukuba, Tsukuba, Japan. Wrote and reviewed the manuscript.; f Toru Yanagawa Professor, Department of Oral and Maxillofacial Surgery, Institute of Clinical Medicine, Faculty of Medicine, and Department of Oral and Maxillofacial Surgery, Ibaraki Prefectural Central Hospital, Tsukuba, Japan. Study concept, wrote and reviewed the manuscript.

**Keywords:** cardiovascular surgery, length of hospital stay, oral care, preoperative oral function management, postoperative oral function management, thermoregulation.

## Abstract

**Purpose:**

This study compared pre- and postoperative interventions on perioperative oral function management during cardiovascular surgery to clarify their effects on patient outcomes.

**Materials and Methods:**

243 consecutive patients who underwent cardiovascular surgery at a single-center facility between January 2018 and December 2021 were retrospectively analyzed. Fever of 38°C or higher was analyzed by univariate and multivariate binary logistic regression analyses, and the length of hospital stay was analyzed using the log-rank test and Cox proportional hazards model.

**Results:**

Patients who received preoperative oral function management were more likely to have a fever than those who received postoperative management. The log-rank test showed a shorter length of hospital stay by 5 days for the preoperative oral function management group; however, the length of hospital stay was not statistically significantly different between the group that underwent postoperative oral function management and the group that did not.

**Conclusion:**

Preoperative oral management is important to reduce the length of hospital stay after cardiovascular surgery.

Factors that define the length of hospital stay after cardiovascular surgery include patient background (pre-existing pneumonia, severe chronic obstructive pulmonary disease, chronic renal failure requiring dialysis,^27^ left atrial diameter > 4 cm)^2,27^ and postoperative complications (pneumonia, atelectasis, atrial fibrillation, stroke),^35^ postoperative hypothermia, bacterial translocation due to bacteria and toxins, and intestinal mucosal atrophy. However, preventing these factors is challenging, thus affecting the ability to shorten a patient’s length of hospital stay. Oral care has been reported to reduce the risk of pneumonia in facilities for older adults with an average age of 82 years.^37^ One of the causes of aspiration pneumonia is an increase in bacterial counts due to decreased salivary secretion, and perioperative management of oral function^4,36^ can be expected to reduce bacterial counts.^26^ Moreover, perioperative oral care has been reported to reduce the risk of postoperative pneumonia in esophageal cancer surgery.^30^ In colorectal cancer surgery, perioperative oral function management has been reported to reduce the risk of surgical site infection and shorten postoperative hospital stay.^23^ The prevalence of postoperative pneumonia is increased in patients with few remaining teeth before cardiovascular surgery.^25^ However, a systematic review revealed that it is unclear whether dental treatment before cardiovascular surgery prevents postoperative complications.^17^ In addition, early postoperative enteral nutrition^14^ and physical therapy, such as respiratory muscle training,^7^ contribute to shorter hospital stays. However, enteral nutrition remains a risk factor for ventilator-associated pneumonia.^11^ Preoperative warming with a warm-air-heating device is expected to be effective^18^ in treating postoperative hypothermia. Several postoperative complications are associated with thermoregulation, and many findings exist regarding the perioperative management of oral function during surgery for malignant tumors. However, there are few reports on the effects of perioperative oral function management in cardiovascular surgery. Furthermore, it is unclear whether perioperative oral function management before or after surgery is associated with abnormal thermoregulation and the length of hospital stay.

Therefore, this study compared preoperative and postoperative interventions on perioperative oral function management cardiovascular surgery to clarify their effects on patient outcomes.

## MATERIALS AND METHODS

### Study Design

To investigate the factors affecting postoperative fever after cardiac surgery, patients’ age, sex, body mass index (BMI), diabetes status, smoking history, surgery duration, blood loss, transfusion volume, pre- and postoperative white blood cell (WBC) count, pre- and postoperative C-reactive protein (CRP), pre- and postoperative hemoglobin (Hb) concentrations, and perioperative oral function management were assessed. In addition, the effects of preoperative and postoperative perioperative oral management on the length of hospital stay were retrospectively recorded.

### Patients

All 243 patients who underwent cardiovascular surgery and visited the Department of Cardiovascular Surgery (single institution) at Ibaraki Prefectural Central Hospital between January 2018 and December 2021 were followed-up and registered. The patient breakdown is shown in Tables A1 and A2.

### Oral Function Management Procedures and Contents

First, a general dentist will develop a perioperative oral function management plan. Perioperative and other oral function management procedures followed methods widely used in Japan.^1,5,37^ Specifically, based on the perioperative oral function management plan, perioperative oral function management I and II are carried out for patients undergoing surgery. Perioperative oral function management I refers to management in which private practice dentists, in-house dentists, and dental hygienists work together to evaluate oral function primarily before and after hospitalization. Perioperative oral function management II refers to management in which in-house dentists and dental hygienists evaluate oral function during hospitalization. This preoperative oral function management includes the extraction of loose teeth that may cause problems during intubation during surgery, removal of infected areas, toothbrushing instructions, periodontal disease checks, scaling, and achieving a plaque-free environment.

**Fig 1:**
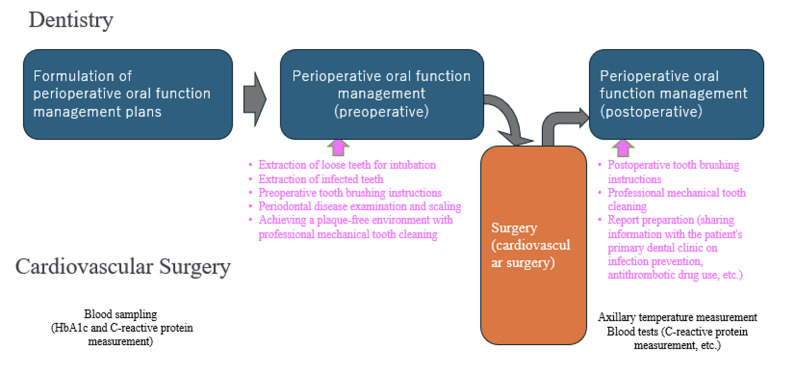
Oral function management schedule. Perioperative oral function management I and II procedures.

The European Society of Cardiology (ESC) guidelines strongly recommend eliminating potential sources of dental sepsis at least 2 weeks before implantation of a prosthetic valve or other intracardiac or intravascular foreign body, unless it is an emergency procedure.^6^ Another study reported a mean waiting period of 20.8 days from tooth extraction to cardiovascular surgery.^19^ Based on these findings, tooth extractions were performed within 2 weeks before surgery for patients who required them (Fig 1). After elective surgery was scheduled, patients were referred to the dental department by the cardiovascular surgery department. First, an orthopantograph was taken and an intraoral examination was performed. A basic periodontal examination was also performed to evaluate periodontal disease. The criteria for extraction were:

Teeth with periodontal pockets of 6 mm or more, or teeth that were mobile not only buccolingually and mesiodistally but also 2 mm or more axially;
Teeth that could not be saved with conservative prosthetic treatment due to caries;
Wisdom teeth that had previously developed pericoronitis;
Teeth that had apical lesions detected by orthopantography and were judged to be unable to be cured with root canal treatment; and teeth that met these criteria were extracted.

Furthermore, preoperative antibiotics (AMPC 2 g) were administered before any invasive procedures (tooth extraction, periodontal treatment) in accordance with the Infective Endocarditis Prevention Guidelines.^21^ Based on studies examining the duration of bacteremia after dental interventions, a minimum waiting period of 24 h after dental prophylaxis (e.g., professional care) is recommended before proceeding with medical or surgical interventions.^31^ The reason for performing preoperative oral function management 24 h before cardiac surgery is to prevent oral plaque from being pushed into the airway during intubation. Therefore, by the morning of the day before surgery, dental hygienists provided patients with oral hygiene instructions and, if necessary, thoroughly removed oral plaque and calculus. In all cases of pre-cardiac surgery oral function management, dental hygienists performed supragingival scaling using an ultrasonic scaler and mechanical tooth surface cleaning. Subgingival scaling and root planing were performed as necessary (Fig 1).

For example, coronary artery bypass surgery for multivessel disease, including acute myocardial infarction associated with diabetes, and artificial vascular replacement for acute aortic root dissection are highly urgent, and preoperative oral function management is not performed in these cases. For emergency surgeries and elective surgeries performed ahead of schedule, oral function management was initiated postoperatively. The group receiving oral care intervention during postoperative intubation was designated as the postoperative oral function management group. Perioperative oral function management was performed by ICU nurses under the guidance of dental hygienists because patients were mechanically ventilated in the intensive care unit. However, mechanical tooth cleaning was not possible in the ICU, so oral care, including swabbing and suctioning of plaque and tongue, was performed three times a day using toothbrushes and sponge brushes. For patients recovering and able to receive treatment at a dental clinic, dental hygienists performed dental cleaning once a week using an ultrasonic scaler. For patients who were unable to complete dental treatment during the perioperative and recovery period after cardiac surgery, tooth extraction and periodontal surgical treatment were performed at each dental outpatient clinic.

### Variables for an Endpoint

Postoperative fever was recorded as a peripheral temperature of 38°C or higher in the axilla three times daily at 7:00 AM, 1:00 PM, and 5:00 PM immediately after surgery and at discharge. Preoperative test data ordered at the cardiovascular surgery outpatient clinic were collected from the most recent data 1 month prior to the surgery date, while postoperative data were obtained from the day after surgery. Preoperative and postoperative CRP measurements were performed using blood samples taken 1 month prior to surgery (the day of the cardiovascular surgery outpatient clinic visit) and within 1 week after surgery (during hospitalization on the ward), respectively. CRP measurements were performed before the completion of oral function management (dental treatment) in all samples. All patients with diabetes had been diagnosed with type 2 diabetes mellitus (T2DM) based on the Japanese Diabetes Diagnostic Criteria^3^ before surgery. Under the Japanese insurance system, HbA1c is only measured once a month, and since it is not possible to measure it frequently, only the blood test results from 1 month before surgery (the day of the cardiovascular surgery outpatient visit) were used. HbA1c was not measured again after surgery.

### Statistical Analysis

All statistical analyses were performed using IBM SPSS Statistics 29.0 (IBM; Armonk, NY, USA), and statistical significance was set at p < 0.05. Univariate and multivariate analyses were performed. A stepwise-reduction binary logistic regression analysis was performed with the presence or absence of a fever of 38°C or higher as the dependent variable. In addition, the relationship between the number of days of hospitalization from surgery to discharge and the presence or absence of preoperative and postoperative perioperative oral function was analyzed using the Kaplan-Meier method (log-rank test) and the Cox proportional hazards model.

### Ethical Approval

The study was conducted in accordance with the Declaration of Helsinki, and all patient information was made unlinkable and anonymized. This study was approved by the Institutional Review Board of Ibaraki Prefectural Central Hospital (1067).

## RESULTS

Univariate analyses evaluating the association between the patient variables and postoperative fever of 38°C or higher showed statistically significant differences in hospital stay (p = 0.04) and postoperative days (p < 0.001) but not in patient age, BMI, pre- and postoperative WBC count, pre- and postoperative CRP, pre- and postoperative Hb, blood transfusion volume, bleeding volume, and operation time (Table 1). No statistically significant differences were found in the univariate analysis of the association between sex, presence of diabetes, smoking history, perioperative oral function management before and after surgery, and type of cardiovascular disease requiring surgery and postoperative fever of 38°C or higher (Table A2). However, in a multivariate logistic analysis, a statistically significant difference was found for perioperative oral function management in the presence of a fever of 38°C or higher (Table 2). Patients with preoperative oral function management were more likely to have fever (odds ratio [OR], 3.71; 95% confidence interval [CI]: 1.039–9.074; p = 0.042), whereas those with postoperative oral function management were less likely to have fever (OR, 0.330; 95% CI, 0.111–0.976; p = 0.045).

**Table 1:** Relationship between continuous variables and presence/absence of fever above 38°C

	Group with fever above 38°C	Group without fever above 38°C	p-value
Age (y)	67.8 ± 10.6	69.9 ± 9.7	0.115
BMI	23.7 ± 4.3	22.8 ± 3.4	0.113
Preoperative WBC (×10^3^/μl)	6.6 ± 2.9	6.1 ± 2.4	0.19
Preoperative CRP (mg/dl)	0.8 ± 1.8	0.6 ± 1.3	0.348
Preoperative Hb (g/dl)	12.7 ± 2.2	12.8 ± 2.1	0.824
Postoperative WBC (×10^3^/μl)	10.2 ± 3.7	10.5 ± 3.2	0.535
Postoperative CRP (mg/d)	6.6 ± 3.5	7.0 ± 3.6	0.463
Postoperative Hb (g/dl)	11.1 ± 1.4	10.9± 1.5	0.312
Blood transfusion volume (ml)	1545.8± 1234.9	1277.4 ± 949.7	0.057
Loss of blood volume (ml)	917.3 ± 1087.0	774.0 ± 781.3	0.262
Surgery duration (hr:min)	5:54 ± 2.03	5:24 ± 1.48	0.057
Length of stay	33.8 ± 28.2	27.9 ± 16.5	0.04
Number of days after surgery	27.2 ± 26.0	19.0 ± 10.2	<0.001
BMI: body mass index; WBC: white blood cell count; CRP: C-reactive protein.			

**Table 2:** Presence or absence of fever (multivariate logistic regression analysis)

	Odds ratio	95% confidence interval	p-value
Surgical time	0.99960	0.999922–0.9999985	0.042
Preoperative oral function management	3.071	1.039–9.074	0.042
Postoperative oral function management	0.330	0.111–0.976	0.045


**Fig 2:**
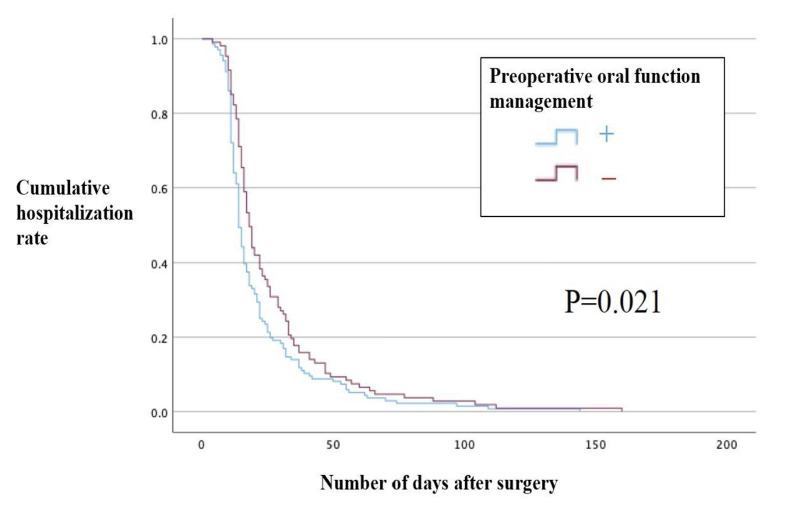
Length of hospital stay in the group that underwent preoperative oral care (Kaplan-Meier method).

**Fig 3:**
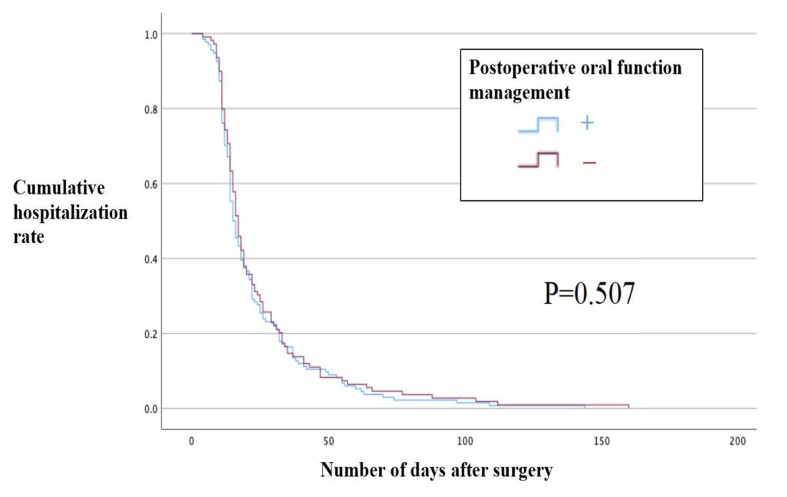
Length of hospital stay in the group that underwent postoperative oral care (Kaplan-Meier method) .

For each group that underwent preoperative and postoperative oral function management, the Kaplan-Meier method and the log-rank test showed that the preoperative oral function management group had a shorter hospital stay by 5 days (p = 0.021, Table 3, Fig 2). However, the length of hospital stay was not statistically significantly different between the group that underwent postoperative oral function management and the group that did not (p = 0.507, Table 3, Fig 3). Furthermore, when the Cox proportional hazards model was examined for the length of hospital stay, hospital stay statistically significantly decreased in the preoperative oral function management group (hazard ratio [HR], 3.705; 95% CI: 2.123–6.466; p < 0.001) but statistically significantly increased in the postoperative oral function management group (HR, 0.291; 95% CI: 0.166–0.510; p < 0.001). Postoperative Hb (mean, 10.990 g/dl) was statistically significantly associated with a shorter hospital stay (HR, 1.110; 95% CI: 1.003–1.229; p = 0.043), and the transfusion group had a statistically significantly longer hospital stay (HR, 0.999554; 95% CI, 0. 999374–0.999735; p < 0.001). However, no statistically significant differences were observed in operative time, blood loss, postoperative white blood cell count, or postoperative CRP level (Table 4).

**Table 3:** Length of hospital stay (log-rank test)

Preoperative oral function management	Average length of hospital stay (days)	95% confidence interval
Postoperative oral function management	Average length of hospital stay (days)	95% confidence interval
+	21.69	18.35–25.03
–	26.65	22.27–31.02
+	23.17	19.74–26.61
–	24.73	20.44–29.03


**Table 4:** Length of stay in hospital (Cox proportional hazards model)

	Hazard ratio	95% confidence interval	p-value
Surgical time	0.999967	0.999945–0.999990	0.004
Blood loss	1.000282	1.000093–1.000470	0.003
Transfusion	0.999554	0.999374–0.999735	<0.001
Postoperative white blood cell count	0.962	0.923–1.003	0.069
Postoperative C-reactive protein	0.961	0.922–1.002	0.064
Postoperative hemoglobin concentration	1.110	1.003–1.229	0.043
Preoperative oral function management	3.705	2.123–6.466	<0.001
Postoperative oral function management	0.291	0.166–0.510	<0.001


## DISCUSSION

Unlike in gastrointestinal surgery, saliva containing oral microorganisms does not flow directly into the surgical field during the perioperative period of cardiovascular surgery. However, thorough preoperative management of oral function is suggested to reduce the risk of postoperative inflammation^22^ and bacteremia,^33^ including ventilator-associated pneumonia,^31^ as well as the frequency of long-term intubation.^20^ Here, the preoperative oral function management group was more prone to fever; however, the length of hospital stay was reduced by 5 days in the preoperative group. In the preoperative management group, mechanical cleaning, removal of subgingival tartar, removal of dental infections, including the extraction of wisdom teeth, and inflammation control were performed adequately. Adequate preoperative oral functional management is assumed to promote immune cell activity and function, causing fever as a physiological response and ultimately shortening the length of hospital stay.

The reason why postoperative oral function management intervention prolongs the hospital stay remained unclear in the present analysis (Table 4). The postoperative oral environment is characterized by decreased saliva production, with the intestinal environment more likely to be disturbed. Thus, bacterial translocation may become heightened, further inducing bacteremia. Therefore, further analysis is needed to elucidate this finding. The oral, nasopharyngeal, and laryngeal environments during and immediately after cardiac surgery differ from those before surgery because of the insertion of an intubation tube. In general, ventilator-associated pneumonia is estimated to occur in 9%–27% of all patients on ventilators,^14^ involving six main organisms (*Staphylococcus aureus* [28.0%], *Pseudomonas aeruginosa* [21.8%], *Klebsiella* [9.8%], *Escherichia coli* [6.9%], *Acinetobacter* [6.8%], and *Enterobacter* [6.3%]). Colonization of upper-respiratory–tract secretions is also a cause of ventilator-associated pneumonia,^13^ where the bacteria are retained in the upper cuff of the endotracheal tube (below the glottis).^8^ Therefore, the upper cuff and pharynx should be suctioned as often as possible. Postoperative care should focus not only on the oral mucosa but also on tongue coating, pharyngolaryngeal mucosa, and intubation-tube contamination, and should be performed thoroughly to avoid the formation of colonies of these organisms. In addition, if pathogenic microorganisms colonize with secretions from the oropharynx and stomach via an underinflated tracheal cuff, they may contribute to the development of ventilator-associated pneumonia.^24^ In actual practice, the cuff pressure is checked to be appropriate, but postoperative mechanical cleaning is difficult in many cases, not only in the upper cuff area. Further, the postoperative course may prolong the period of supine positioning and delay the resumption of oral intake of food and beverages. Therefore, the postoperative management of oral function is limited in terms of cleaning time and position per visit. In particular, postoperative oral care is likely to be inadequate in patients with a poor postoperative course because they cannot move around easily and are prone to malnutrition. Therefore, a toothbrush^28^ with a suction mechanism that prevents the dispersal of dental plaque and collects contaminants from the oral cavity and pharynx may be more suitable for postoperative oral care. In addition, wiping the oral mucosa and tongue and possible suctioning of the upper cuff are desirable to prevent ventilator-associated pneumonia in intubated patients who are edentulous or have few remaining teeth. In other words, hospital stay may have been prolonged owing to multiple factors other than postoperative oral function management alone. Therefore, postoperative efforts are focused on suctioning the pharynx and larynx as much as possible, removing tongue coating, and preventing mucosal dryness. Patients with adequate preoperative oral function management can be transferred to postoperative oral care, and oral hygiene can be maintained after surgery. Conversely, in emergency surgery cases, sufficient preoperative intervention is not possible, and postoperative oral hygiene is difficult to control.

Patients receiving postoperative oral functional management were less likely to develop fever compared to those not having functional oral management. Several possible mechanisms may have contributed to this. During general anesthesia, peripheral blood vessels dilate, and heat is transferred from the center of the body to the periphery; when there is no heat production during the operation, heat dissipation by heat conduction occurs. In addition, endogenous thermogenic substances (IL-1α, IL-1β, and IL-6, tumor necrosis factor α, β) are secreted from the surgical site, which increases the set point of the body temperature center. Therefore, postoperative arousal can lead to thermogenesis (shivering). Similarly, the administration of large amounts of unwarmed fluid, extracorporeal circulation, and massive bleeding during surgery can cause hypothermia. Surgery and anesthesia generally produce immunosuppression in the immediate postoperative period,^10^ and in practice, opportunistic infections caused by herpes viruses (Herpes simplex virus, Varicella zoster virus) and oral and esophageal candidiasis can occur in the postoperative period. Immunocompromised patients often do not develop a fever. The causal relationship between the immune system and thermoregulation^2^ can be reversed. Coronavirus disease and malaria have been reported in patients without fever,^9,16^ but patients with complex immunosuppression require caution. Thus, postoperative management of oral function mobilizes endogenous and exogenous febrile substances, such as endotoxins and peptidoglycans. As a result, postoperative immunosuppression and hypothermia may be promoted, and hospital stays may be prolonged. However, in the present study, the postoperative fever group’s average hospital stay was 5–8 days longer than that of the non-febrile group. The non-febrile group consisted of patients with a good postoperative course and patients who were immunodeficient with prolonged postoperative hypothermia. The febrile group included patients with physiologically reactive fever and fever caused by postoperative complications. Therefore, controlling postoperative complications is important for shortening the length of hospital stay.

The target level for renal anemia is an Hb level of 11 g/dl,^29^ and anemia is a risk factor for cardiovascular disease.^34^ From this perspective, a postoperative Hb level of approximately 11 g/dl reduces the length of hospital stay, which is consistent with the results of prolonged hospital stays in cases that require blood transfusions. Therefore, it is advisable to correct for postoperative Hb level of 11 g/dl, regardless of whether the anemia is dilutionally progressive, with a tendency toward heart failure, iron deficiency anemia, or renal anemia.

### Limitations

Preoperative oral function management decreased postoperative hospital stay. However, as this was a retrospective study, the results were not obtained by randomizing the patients into preoperative or postoperative groups. Therefore, a selection bias could not be avoided. The Japanese medical care reimbursement system is based on a combination of the name of the disease or diagnosis (diagnosis) and the medical treatment provided (procedure). The amount of reimbursement in the diagnosis procedure combination (DPC) system is composed of the piece rate and comprehensive payment. In Japan, reimbursement differs depending on the length of hospital stay; points are added if the patient is discharged earlier than the average length of hospital stay and subtracted if the patient remains longer than the average length of hospital stay. Therefore, some cases may have been arbitrarily determined by the DPC. Nevertheless, this clinical trial is meaningful because it reaffirms the importance of preoperative management of oral function.

## CONCLUSION

Preoperative oral management is important to reduce the length of hospital stay after cardiovascular surgery.

## Appendix

**Table A2:** Relationship between categorical variables and presence/absence of fever above 38°C

		Group with fever above 38°C	Group without fever above 38°C	p-value
Sex	Male	98	66	0.969c
	Female	47	32	
Diabetes mellitus	Yes	51	42	0.230F
	No	94	56	
Smoking	Yes	87	59	0.975c
	No	58	39	
Preoperative oral function management	Yes	78	58	0.406c
	No	67	40	
Postoperative oral function management	Yes	81	53	0.784c
	No	64	45	
Type of cardiovascular diseases that lead to surgery	Large vessel disease such as aortic dissection	22	8	0.165c
	Valvular disease	87	69	
	Ischemic heart disease	36	21	
c: chi-squared test; F: Fisher’s exact test				

**Table A1:** Preoperative clinical characteristics of patients (N = 243)

	Average ± SD
Age, years	68.7 ± 10
Height	161 ± 3.5 cm
Body weight	61 ± 4.5 kg
BMI	23.3 ± 3.9


## References

[ref1] Ames NJ, Sulima P, Yates JM, McCullagh L, Gollins SL, Soeken K (2011). Effects of systematic oral care in critically ill patients: a multicenter study. Am J Crit Care.

[ref2] Appenheimer MM, Evans SS (2018). Temperature and adaptive immunity. Handb Clin Neurol.

[ref3] Araki E, Goto A, Kondo T, Noda M, Noto H, Origasa H (2020). Japanese clinical practice guideline for diabetes 2019. Diabetol Int.

[ref4] Bergan EH, Tura BR, Lamas CC (2014). Impact of improvement in preoperative oral health on nosocomial pneumonia in a group of cardiac surgery patients: a single arm prospective intervention study. Intensive Care Med.

[ref5] Chalmers JM, King PL, Spencer AJ, Wright FA, Carter KD (2005). The oral health assessment tool-validity and reliability. Aust Dent J.

[ref6] Delgado V, Ajmone Marsan N, de Waha S, Bonaros N, Brida M, Burri H (2023). 2023 ESC Guidelines for the management of endocarditis: Developed by the task force on the management of endocarditis of the European Society of Cardiology (ESC) Endorsed by the European Association for Cardio-Thoracic Surgery (EACTS) and the European Association of Nuclear Medicine (EANM). Eur Heart J.

[ref7] Elkins M, Dentice R (2015). Inspiratory muscle training facilitates weaning from mechanical ventilation among patients in the intensive care unit: a systematic review. J Physiother.

[ref8] Frost SA, Azeem A, Alexandrou E, Tam V, Murphy JK, Hunt L (2013). Subglottic secretion drainage for preventing ventilator associated pneumonia: a meta-analysis. Aust Crit Care.

[ref9] Gadient HS, Müller DJ, Chaney M, Blum J (2015). No fever, no malaria? A diagnostic challenge in an immunocompromised patient. Travel Med Infect Dis.

[ref10] Hogan BV, Peter MB, Shenoy HG, Horgan K, Hughes TA (2011). Surgery induced immunosuppression. Surgeon.

[ref11] Ogawa M, Satomi-Kobayashi S, Yoshida N, Tsuboi Y, Komaki K, Nanba N (2020). Impact of oral health status on postoperative complications and functional recovery after cardiovascular surgery. CJC Open.

[ref12] Ibrahim KS, Kheirallah KA, Al Manasra ARA, Megdadi MA (2024). Factors affecting duration of stay in the intensive care unit after coronary artery bypass surgery and its impact on in-hospital mortality: a retrospective study. J Cardiothorac Surg.

[ref13] Jones RN (2010). Microbial etiologies of hospital-acquired bacterial pneumonia and ventilator-associated bacterial pneumonia. Clin Infect Dis 2010;51(suppl 1): S81–87. Erratum in: Clin Infect Dis.

[ref14] Kalanuria AA, Ziai W, Mirski M (2014). Ventilator-associated pneumonia in the ICU. Crit Care.

[ref15] Kamei H, Hachisuka T, Nakao M, Takagi K (2005). Quick recovery of serum diamine oxidase activity in patients undergoing total gastrectomy by oral enteral nutrition. Am J Surg.

[ref18] Melling AC, Ali B, Scott EM, Leaper DJ (2001). Effects of preoperative warming on the incidence of wound infection after clean surgery: a randomised controlled trial. Lancet.

[ref19] Miao XZ, Christopher FV, Ondřej H, Sarah KL, Kyle WK, Thomas JS (2024). Oral Health Clearance Outcomes for Cardiovascular Surgery. Mayo Clin Proc Innov Qual Outcomes.

[ref20] Motoi T, Matsumoto K, Imoto Y, Oho T (2022). Perioperative oral management prevents complications of heart valve surgery. Int Dent J.

[ref21] Nakatani S, Ohara T, Ashihara K, Izumi C, Iwanaga S, Eishi K (2019). JCS 2017 guideline on prevention and treatment of infective endocarditis. Circ J.

[ref22] Nishi H, Takahashi S, Ohta K, Takamoto M, Shigeishi H, Go S (2021). Effects of perioperative oral care on postoperative inflammation following heart valve surgery. Oral Dis.

[ref23] Nobuhara H, Yanamoto S, Funahara M, Matsugu Y, Hayashida S, Soutome S (2018). Effect of perioperative oral management on the prevention of surgical site infection after colorectal cancer surgery: a multicenter retrospective analysis of 698 patients via analysis of covariance using propensity score. Medicine (Baltimore).

[ref24] Nseir S, Gaudet A (2021). Continuous control of tracheal cuff pressure and ventilator-associated pneumonia: beyond agate and feng shui. Chest.

[ref25] Hunter JD (2006). Ventilator associated pneumonia. Postgrad Med J.

[ref26] Osako R, Matsuda Y, Itohara C, Sukegawa-Takahashi Y, Sukegawa S, Okuma S (2021). Relationship between oral bacterial count and postoperative complications among patients with cardiovascular disease treated by surgery: a retrospective cohort study. Healthcare (Basel).

[ref28] Schleder B, Stott K, Lloyd RC (2002). The effect of a comprehensive oral care protocol on patients at risk for ventilator-associated pneumonia. J Advocate Health Care.

[ref29] Singh AK, Szczech L, Tang KL, Barnhart H, Sapp S, Wolfson M (2006). Correction of anemia with epoetin alfa in chronic kidney disease. N Engl J Med.

[ref31] Souza A, Rocha AL, Castro WH, Gelape CL, Nunes MCP, SR Oliveir (2017). Dental management for patients undergoing heart valve surgery. J Card Surg.

[ref32] Stonecypher K (2010). Ventilator-associated pneumonia: the importance of oral care in intubated adults. Crit Care Nurs Q.

[ref33] Suenaga H, Schifter M, Chen N, Ali F, Byth K, Peck C (2023). Impact of oral/dental disease burden on postoperative infective complications: a prospective cohort study. Clin Oral Investig.

[ref34] Vlagopoulos PT, Tighiouart H, Weiner DE, Griffith J, Pettitt D, Salem DN (2005). Anemia as a risk factor for cardiovascular disease and all-cause mortality in diabetes: the impact of chronic kidney disease. J Am Soc Nephrol.

[ref35] Weintraub WS, Jones EL, Craver J, Guyton R, Cohen C (1989). Determinants of prolonged length of hospital stay after coronary bypass surgery. Circulation.

[ref36] Yasny J (2010). The importance of oral health for cardiothoracic and vascular patients. Semin Cardiothrorac Vasc Anesth.

[ref37] Yoneyama T, Yoshida M, Matsui T, Sasaki H (1999). Oral care and pneumonia. Oral care working group. Lancet.

